# HCV NS3 protease enhances liver fibrosis via binding to and activating TGF-β type I receptor

**DOI:** 10.1038/srep03243

**Published:** 2013-11-22

**Authors:** Kotaro Sakata, Mitsuko Hara, Takaho Terada, Noriyuki Watanabe, Daisuke Takaya, So-ichi Yaguchi, Takehisa Matsumoto, Tomokazu Matsuura, Mikako Shirouzu, Shigeyuki Yokoyama, Tokio Yamaguchi, Keiji Miyazawa, Hideki Aizaki, Tetsuro Suzuki, Takaji Wakita, Masaya Imoto, Soichi Kojima

**Affiliations:** 1 Micro-signaling Regulation Technology Unit, RIKEN Center for Life Science Technologies, Saitama 351-0198, Japan; 2 Department of Biosciences and Informatics, Faculty of Science and Technology, Keio University, Kanagawa 223-8522, Japan; 3 Drug Discovery Laboratory, Wakunaga Pharmaceutical Co., Ltd., Hiroshima 739-1195, Japan; 4 RIKEN Systems and Structural Biology Center, Kanagawa 230-0045, Japan; 5 RIKEN Structural Biology Laboratory, Kanagawa 230-0045, Japan; 6 Department of Virology II, National Institute of Infectious Diseases, Tokyo 162-8640, Japan; 7 Division of Structural and Synthetic Biology, RIKEN Center for Life Science Technologies, Kanagawa 230-0045, Japan; 8 Department of Biochemistry, Interdisciplinary Graduate School of Medicine and Engineering, University of Yamanashi, Yamanashi 409-3898, Japan; 9 Department of Laboratory Medicine, the Jikei University School of Medicine, Tokyo 105-8461, Japan; 10 RIKEN Program for Drug Discovery and Medical Technology Platforms, Saitama 351-0198, Japan; 11 Department of Infectious Diseases, Hamamatsu University School of Medicine, Shizuoka 431-3192, Japan

## Abstract

Viruses sometimes mimic host proteins and hijack the host cell machinery. Hepatitis C virus (HCV) causes liver fibrosis, a process largely mediated by the overexpression of transforming growth factor (TGF)-β and collagen, although the precise underlying mechanism is unknown. Here, we report that HCV non-structural protein 3 (NS3) protease affects the antigenicity and bioactivity of TGF-β2 in (CAGA)_9_-Luc CCL64 cells and in human hepatic cell lines via binding to TGF-β type I receptor (TβRI). Tumor necrosis factor (TNF)-α facilitates this mechanism by increasing the colocalization of TβRI with NS3 protease on the surface of HCV-infected cells. An anti-NS3 antibody against computationally predicted binding sites for TβRI blocked the TGF-β mimetic activities of NS3 *in vitro* and attenuated liver fibrosis in HCV-infected chimeric mice. These data suggest that HCV NS3 protease mimics TGF-β2 and functions, at least in part, via directly binding to and activating TβRI, thereby enhancing liver fibrosis.

Viruses sometimes take over the host cell machinery by mimicking host cell proteins. This strategy infers survival, infection, and replication advantages to the virus^1^,^2^, which may thereby contribute to the development of human disease.

Chronic hepatitis C virus (HCV) infection is one of the major causes of liver fibrosis, cirrhosis, and hepatocellular carcinoma^3^,^4^. However, the molecular mechanism by which HCV induces liver fibrosis is not fully understood. An estimated 130–170 million people worldwide are infected with HCV^5^. HCV, classified in the genus *Hepacivirus* of the family *Flaviviridae*, is a positive-strand RNA virus with an approximately 9.6-kb viral genome encoding structural (core, E1, and E2) and non-structural proteins (p7, NS2, NS3, NS4A, NS4B, NS5A, and NS5B)^6^. Of these proteins, NS3 is a member of the serine protease family that cleaves the HCV polyprotein to generate mature viral proteins that are required for viral replication^7^.

Liver fibrosis, a common feature of chronic liver diseases, is caused by the excessive accumulation of extracellular matrix (ECM) proteins, including collagen. Transforming growth factor (TGF)-β, the most potent fibrogenic cytokine, is produced in its high molecular weight latent form and partly activated through the proteolytic cleavage of its propeptide region, termed latency associated protein (LAP), by serine proteases, plasmin, and plasma kallikrein^8^,^9^. The resultant active TGF-β signals via TGF-β type I (TβRI) and type II receptors (TβRII), inducing the phosphorylation of Smad2/3, which then binds to Smad4 and forms a complex that enters the cell nucleus. This complex acts as a transcription factor that controls the expression of target genes, including collagen and TGF-β itself, by binding to the DNA elements containing the minimal Smad-binding element, CAGA box^10^.

Because the LAPs of TGF-β2 and -β3 have sequences that share partially homology with the NS3 cleavage site between NS3 and NS4A of HCV^7^, we speculated that NS3 might activate TGF-β2 and/or TGF-β3 via the proteolytic cleavage of their LAP portions. We found, however, that NS3 protease DID NOT directly activate latent TGF-β2/3. Instead, it mimicked TGF-β2 and induced TGF-β signaling by binding and activating TβRI, leading to the induction of fibrogenic genes. This pathway was enhanced in the presence of an inflammatory cytokine, tumor necrosis factor (TNF)-α, as TNF-α increased the expression of TβRI. Furthermore, we found that NS3 colocalized with TβRI on the surface of an HCV-infected hepatoma cell line, and we observed direct binding between recombinant NS3 and TβRI. These phenomena were reproduced in chimeric mice transplanted with human hepatocytes that had been infected with HCV. These data suggest a novel mechanism by which HCV induces liver fibrosis.

## Results

### HCV NS3 protease exerted TGF-β mimetic activity via TβRI

**Figure 1:**
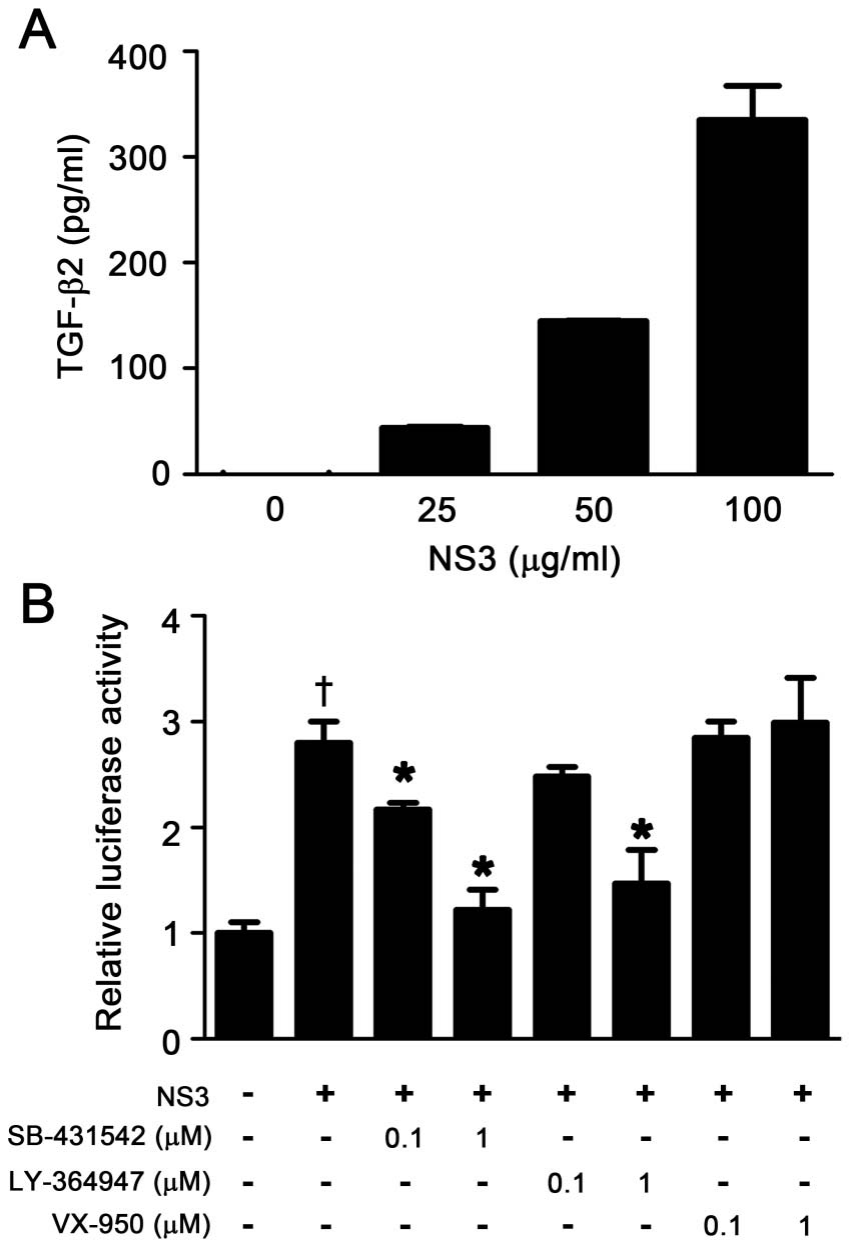
HCV NS3 protease exerted TGF-β mimetic activity via the type I receptor. (A) TGF-β2 antigenicity of NS3. The indicated concentrations of recombinant NS3 protease were used in the TGF-β2 ELISA assays. (B) TGF-β mimetic activity of NS3 and its suppression by TβRI kinase inhibitors. (CAGA)_9_-Luc CCL64 cells were stimulated with 100 μg/ml of recombinant NS3 protease for 24 hours, with or without the indicated concentration of TβRI kinase inhibitor or the NS3 protease inhibitor VX-950 (telaprevir). After 24 hours, the cells were harvested and luciferase activity measured. †*p* < 0.05 compared with untreated control cells, *p < 0.05 compared with NS3-treated cells without any inhibitors. The data are shown as the mean ± SD (n = 3), and representative results from three independent experiments with similar results are shown.

To confirm whether HCV NS3 protease might induce the activation of latent TGF-β2, bacterially expressed recombinant NS3 (Supplementary Fig. S1) was incubated with conditioned medium obtained from HEK293T cells transiently overexpressing latent TGF-β2, and the concentration of active TGF-β2 in the reaction mixtures were measured by ELISA. Although the addition of NS3 increased active TGF-β2 concentrations in a dose-dependent manner, these increases were not time-dependent (Supplementary Fig. S2). Instead, we found that NS3 protease itself reacted with TGF-β2 in a dose-dependent manner, as determined by ELISA (Fig. 1A). Next, to assess whether NS3 could induce the bioactivity of TGF-β via TβRI, and whether its activity was dependent on protease activity, we performed a luciferase reporter assay with the TGF-β-responsive (CAGA)_9_-Luc reporter in CCL64 cells. NS3 demonstrated TGF-β mimetic activity, which was alleviated in the presence of TβRI kinase inhibitors (SB-431542 and LY-364947) in a dose-dependent manner (Fig. 1B). In contrast, an NS3 protease inhibitor, VX-950 (telaprevir), did not affect luciferase activity (Fig. 1B). An unrelated protein with almost the same molecular weight as NS3, HLA class II histocompatibility antigen, DM α chain (HLA-DMA), as well as a carrier-free, tag-control sample, did not exert TGF-β mimetic activity, thus demonstrating the specificity of NS3 (Supplementary Fig. S3). Additionally, an anti-TGF-β2 antibody that detected NS3 in the TGF-β2 ELISA did not inhibit luciferase activity (Supplementary Fig. S4).

### NS3 stimulated collagen production in hepatic cells, which was augmented by TNF-α

**Figure 2:**
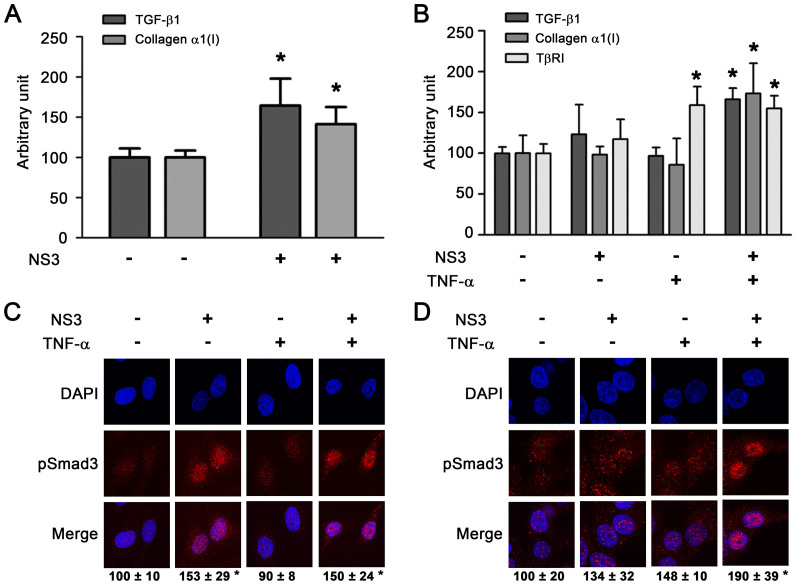
Cooperativity between NS3 and TNF-α in the stimulation of TGF-β1, collagen α1(I), and TβRI expression. (A) Effect on TGF-β1 and collagen α1(I) mRNA expression in LX-2 cells. The cells were stimulated with 50 μg/ml of NS3 for 12 hours. Total cellular RNA was isolated and reverse transcribed to cDNA, and real-time PCR was performed as described in the Methods section. *p < 0.05 compared with untreated control cells. (B) Effect of pretreatment with TNF-α on the stimulation of expression of TGF-β1, collagen α1(I), and TβRI by NS3 protease in HC cells. Following the pretreatment of the cells with 20 ng/ml TNF-α for 12 hours, they were stimulated with 25 μg/ml NS3 for 12 hours, and mRNA expression was measured as described above. *p < 0.05 compared with untreated cells. The data are shown as the mean ± SD (n = 3). (C and D) The effect of pretreatment with TNF-α on the stimulation of phosphorylation of Smad3 by NS3 protease in LX-2 cells (C) and Hc cells (D). After the cells were treated with 20 ng/ml TNF-α for 12 hours and 25 μg/ml NS3 for another 12 hours, they were fixed, and immunofluorescent staining was performed as described in the Methods section. The experiments were performed in duplicate. The relative fluorescence intensities of phospho-Smad3 (% of untreated control cells) in 4 randomly selected fields from each dish were calculated with ZEN software and are shown as the mean ± SD. The results are representative of three independent experiments with similar results.

We examined the effect of NS3 on the expression of TGF-β1 and collagen α1 (I) in the human hepatic stellate cell line LX-2. Treatment with NS3 for 12 hours significantly increased both TGF-β1 (1.6-fold) and collagen α1 (I) (1.4-fold) expression in these cells (Fig. 2A). On the contrary, NS3 did not affect the expression of these genes in the normal hepatic cell line Hc. The pretreatment of the cells with tumor necrosis factor-α (TNF-α) enhanced increased TGF-β1 and collagen α1 (I) expression mediated by NS3 and was also accompanied by an increase in TGF-β receptor expression (Fig. 2B). Further increases in TβRI expression were not observed by combination treatment with TNF-α, suggesting that TNF-α increased TβRI expression, which may have enhanced the TGF-β mimetic activity of NS3 in these cells. Furthermore, Smad3 phosphorylation was also induced by NS3 in Hc cells that had been pretreated with TNF-α (Fig. 2D). A similar cooperativity between TNF-α and NS3 protease was not observed in LX-2 cells (Fig. 2C).

### Interaction between NS3 and TβRI on the surface of HCV-infected HCC cells

**Figure 3:**
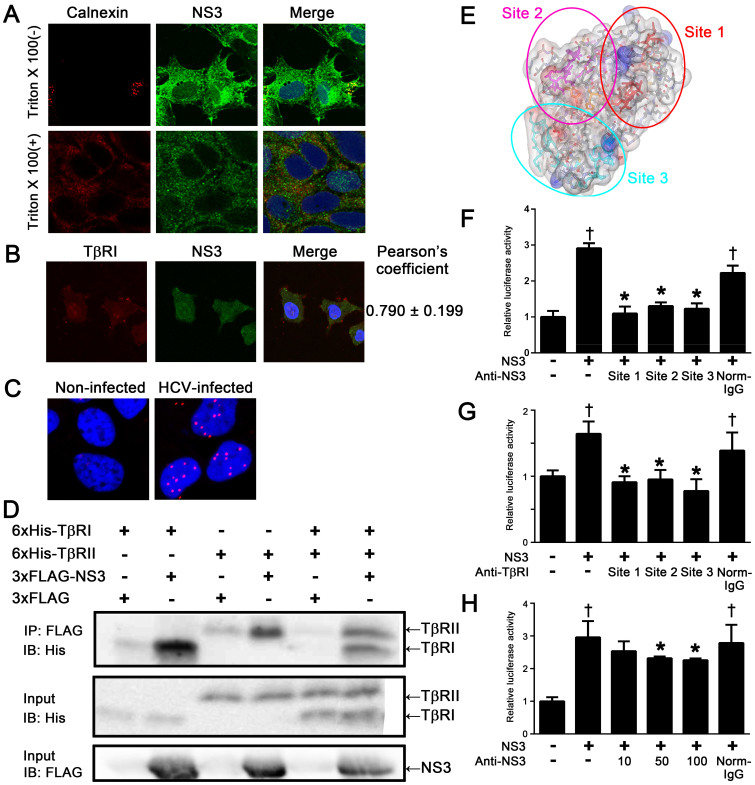
NS3 protease colocalized and directly interacted with TβRI on the surface of HCV-infected cells. (A) The detection of NS3 protease on the surface of HCV-infected Huh-7.5.1 cells. The cells were fixed, followed ± by permeabilization with Triton-X 100, and then stained with DAPI, anti-NS3 antibody, and anti-calnexin antibody. (B) The colocalization of NS3 protease with TβRI in HCV-infected Huh7.5.1 cells. The cells were fixed and stained with DAPI, anti-NS3 antibody, and anti-TβRI antibody, as described in the Methods section. Pearson's colocalization coefficient values were obtained from 4 randomly selected fields using the ZEN software. The results are shown as the mean ± SD and are representative of three independent experiments with similar results. (C) The detection of NS3-TβRI proximity by in situ PLA in HCV-infected Huh-7.5.1 cells. The red dots indicate interactions between NS3 protease and TβRI, and the nuclei were identified by DAPI staining. (D) The physical interaction of NS3protease with TβRI and TβRII. FLAG-tagged NS3protease was incubated with 6xHis-tagged TβRI and/or TβRII and immunoprecipitated. The coprecipitated proteins were visualized by immunoblotting using anti-His antibody. The gels were run under the same experimental conditions. Cropped blots are shown (full-length blots are presented in Supplementary Fig. S5). (E) The structural overview of the NS3protease. The indicated colored amino acids (site 1, red; site 2, magenta; and site 3, cyan) show the important residues within the putative binding sites to TβRI, and the sequences are presented in Table 1. TGF-β mimetic activity of NS3 was inhibited in the presence of either anti-NS3 polyclonal antibodies against the predicted binding sites of TβRI (F), or anti-TβRI polyclonal antibodies against predicted binding sites of NS3 (G), and anti-NS3 monoclonal antibody against predicted binding site 3 of TβRI (H). Luciferase activities in (CAGA)_9_-Luc CCL64 cells were measured as before. Normal mouse IgG (Norm-IgG) was used as a negative control. The data are shown as the mean ± SD. †*p* < 0.05 compared with untreated control cells, *p < 0.05 compared with NS3-treated cells without any antibodies. Representative results from three independent experiments with similar results are shown.

NS3 was immunostained on the surface of HCV-infected Huh-7.5.1 cells both with and without permeabilization. In contrast, an ER marker, calnexin, was only positive after the permeabilization of the cells (Fig. 3A). To examine whether NS3 that was localized to the surface of HCV-infected Huh-7.5.1 cells interacted with TβRI, we performed co-immunostaining (Fig. 3B) and in situ proximity ligation assay (PLA) (Fig. 3C) using antibodies against NS3 and TβRI. Both results showed that NS3 was colocalized and formed a complex with TβRI on the cell surface. Because LX-2 cells (hepatic stellate cells) are not infected with HCV, the data were not recorded. We also co-cultured Huh-7.5.1 infected with HCV and LX-2 cells and examined them using *in situ* PLA. However, the interaction between NS3 protease and TβRI was not observed on the surface of LX-2 cells. Furthermore, we performed co-immunoprecipitation assays using recombinant NS3 and the extracellular domain of TβRI and TβRII. As shown in Figure 3D, FLAG-tagged NS3 bound to TβRI and TβRII, whereas FLAG-tag alone failed to interact with TGF-β receptors (Fig. 3D and Supplementary Fig. S5).

Docking simulation using the Katchalski-Katzir algorithm predicted that NS3 interacts with TβRI at three sites, T22-S42, T76-P96, and G120-S139, in NS3 and F55-M70, I72-V85, and C86-Y99 in TβRI, respectively (Fig. 3E, Table 1, and Supplementary Fig. S6). The predicted binding site peptides, particularly the peptide derived from site 3, completely blocked the interaction between NS3 and TβRI in the immunoprecipitation experiment (Supplementary Fig. S7A). Antibodies produced to these predicted binding sites within both NS3 and TβRI decreased the TGF-β mimetic activity of NS3 in (CAGA)_9_-Luc CCL64 cells (Fig. 3F–H). Furthermore, the anti-NS3 antibody inhibited HCV-induced Smad3 phosphorylation (Supplementary Fig. S7B).

### Anti-NS3 antibody prevented liver fibrosis in HCV-infected chimeric mice

**Figure 4:**
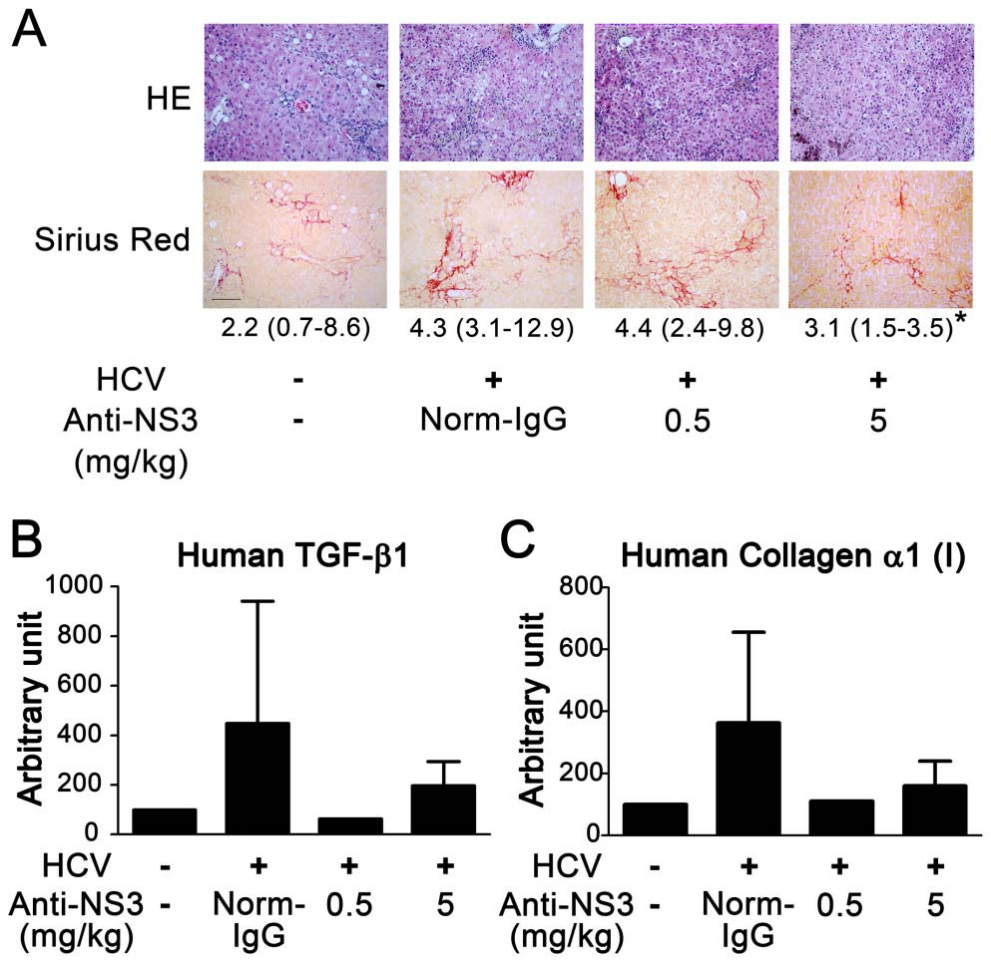
Anti-NS3 antibody attenuated liver fibrosis in the HCV-infected chimeric mice. (A) Staining of liver sections. Paraffin sections were prepared from the livers of HCV-infected chimeric mice 16 weeks after HCV inoculation, and stained with hematoxylin and eosin (upper panels) and Sirius Red (lower panels). An anti-NS3 antibody was administered at the indicated doses, and normal mouse IgG (Norm-IgG) was administered at a dose of 5 mg/kg. For each group, the median ratios in Sirius Red positive/total area (%) from 6 randomly selected fields are shown, with the range in parentheses. *p < 0.05 compared with HCV-infected mice without anti-NS3 antibody. Scale bar = 100 μm. The representative result from 6 randomly selected fields is shown. (B) and (C) Hepatic mRNA expression in HCV-infected chimeric mice. Total RNA was isolated from the livers of these mice and reverse transcribed to cDNA, and real-time PCR was performed as described in the Methods section to quantitate the expression of human TGF-β1 expression (B) and human collagen α1 (I) (C). The data are shown as the mean ± SD, and representative results from two independent experiments with similar results are shown.

To test our hypothesis that NS3 exerts TGF-β mimetic activity, thereby causing liver fibrosis, we examined whether the anti-NS3 antibody could prevent liver fibrosis in HCV-infected human hepatocyte-transplanted chimeric mice. The anti-NS3 antibody significantly prevented hepatic collagen accumulation in the mice (Fig. 4A) and decreased the mRNA expression of both TGF-β1 and collagen α1 (I) (Fig. 4B and 4C). There was no significant change in the serum levels of human albumin and HCV RNA during treatment with the anti-NS3 antibody (Supplementary Fig. S8A and S8B).

## Discussion

Several groups have studied the molecular mechanisms by which HCV induces liver fibrosis and have reported the following: (i) HCV core protein activates the TGF-β1 promoter via the MAPK pathway in core protein-expressing human hepatocellular carcinoma HepG2 cells^11^; (ii) recombinant core protein upregulates the expression of fibrogenic genes in the human hepatic stellate cell line LX-2 via the toll-like receptor 2 12 and the obese receptor^13^; and (iii) NS3 protease induces TGF-β1 production in NS3-overexpressing human hepatoma Huh-7 cells^14^. Our data show that NS3 protease mimics TGF-β2 and directly exerts its activity, at least in part, via binding to and activating TβRI, thereby enhancing liver fibrosis. The following experiments should be carried out in the future: effect of NS3 on TβRI phosphorylation, the expression of TGF-β2, TGF-β3, and other TGF-β responsive genes, such as plasminogen activator inhibitor-1, a tissue inhibitor of metalloproteinase-1, and α-smooth muscle actin, to further validate the TGF-β mimetic activity of NS3.

HCV NS3 is a chimera of a helicase and serine protease, which cleaves not only the junction between NS3-4A, NS4A-4B, NS4B-5A, and NS5A-5B for viral polyprotein processing, which is essential to the viral lifecycle, but also the toll-interleukin-1 receptor domain-containing, adaptor-inducing beta interferon, and mitochondrial antiviral signaling protein, which results in the disruption of innate immune responses^7^,^15^. An NS3 protease inhibitor, telaprevir, which was approved by the FDA in 2011, has been used in triple combination therapy with the current standard treatment of PEGylated interferon and ribavirin^16^. Telaprevir did not inhibit TGF-β mimetic activity in a (CAGA)_9_-Luc reporter gene assay (Fig. 1C), suggesting that the TGF-β mimetic activity of NS3 is independent of its protease activity.

Much interest has centered on the fact that extraordinarily high concentrations of NS3 protease, up to 100 μg/ml, could exist in proximity to a TGF-β receptor. This line of inquiry led us to identify the cooperativity between NS3 and TNF-α, although the cooperative effect was maximal at one fourth this concentration of NS3. Serum levels of TNF-α in chronic hepatitis C patients are known to be significantly higher than those in healthy subjects^17^,^18^. We showed that TNF-α increased the susceptibility of cells to NS3 by enhancing the expression of TβRI, thereby further increasing the levels of profibrogenic genes (Fig. 2B). Various hepatic cell lines expressed different levels of TβRI, and there appeared to be a threshold in the level of TβRI that enabled cells to produce collagen mRNA upon stimulation with NS3. In particular, Hc cells expressed levels of TβRI below this predicted threshold (Supplementary Fig. S9). Consistent with our findings, carbon tetrachloride has recently been reported to induce acute liver injury, specifically significant liver fibrosis with inflammation, in transgenic mice expressing the full-length HCV polyprotein^19^.

We documented the colocalization of NS3 and TβRI on the cell surface of HCV JFH-1-infected Huh-7.5.1 cells (Fig. 3). The results of co-immunoprecipitation and in situ PLA studies supported this conclusion. In future studies, we intend to use mutagenesis experiments of the predicted binding site and competition assays using NS3 and TGF-β in (CAGA)_9_-Luc CCL64 cells to determine the mechanism of NS3 and TβRI binding. However, at present, how NS3 is released to the extracellular milieu remains to be elucidated. One possibility is that NS3 leaks passively from injured hepatocytes, as is the case for alanine aminotransferase and aspartate aminotransferase. Another possibility is that NS3 is secreted from HCV-infected cells via the Golgi complex. A recent report showed that nonstructural protein (NS) 1 of the dengue virus (DENV) and West Nile virus (WNV) is secreted from DENV- and WNV-infected cells through the Golgi complex following expression in association with the endoplasmic reticulum. Like HCV, these viruses are also members of the family *Flaviviridae*^20^.

Zhang et al.^21^ identified antibodies against NS3 in the serum of chronic hepatitis C patients and suggested that extracellular NS3 may be present in such cases. However, it remains unclear whether the concentration of HCV NS3 is as high as in our in vitro experiments. Although DENV NS1 has been reportedly detected at high levels (up to 50 μg/ml) in the serum of DENV-infected patients^22^, further study is warranted to determine the serum or tissue NS3 concentrations in patients with chronic hepatitis C.

In this study, we generated polyclonal and monoclonal anti-NS3 antibodies that block the NS3-TβRI interaction. All anti-NS3 and anti-TβRI polyclonal antibodies generated against the predicted binding sites almost completely blocked TGF-β mimetic activity. This finding was likely due to steric hindrance by these antibodies or a requirement of binding at all three sites for signal transduction by NS3. The monoclonal antibody is a powerful tool that can be used to explore our working hypothesis that NS3 enhances liver fibrosis via the TGF-β receptor *in vivo*. We showed that the anti-NS3 monoclonal antibody generated against a predicted binding site to TβRI ameliorated liver fibrosis in HCV-infected human hepatocyte transplanted chimeric mice (Fig. 4A–C). The control of fibrosis after the eradication of the virus determines the prognosis, including the likelihood of progression to tumorigenesis. Therefore, the NS3 antibody against the TβRI binding site might have a clinical benefit in HCV patients with cirrhosis after combination therapy.

In conclusion, we demonstrated for the first time that HCV NS3 protease serves as a novel TGF-β receptor ligand and enhances liver fibrosis. This phenomenon might be beneficial to the virus, as TGF-β signals suppress host immunity. Our results provide elucidation regarding the molecular mechanism by which HCV induces liver fibrosis.

## Methods

### Materials

SB-431542 and LY-364947 were purchased from Sigma-Aldrich (St. Louis, MO). Recombinant human TNF-α was purchased from R&D systems, Inc. (Minneapolis, MN). Anti-NS3 antibody and anti-calnexin antibody were purchased from Abcam (Cambridge, UK). Anti-TβRI antibody and anti-phospho-Smad3 antibody were purchased from Santa Cruz Biotechnology (Santa Cruz, CA) and Immuno-Biological Laboratories (Gunma, Japan), respectively. Anti-Flag M2 antibody and anti-His antibody were purchased from Sigma (St. Louis, MO). Anti-NS3 antibodies and anti-TβRI antibodies against predicted binding sites were provided by the BioMatrix Research Institute (Chiba, Japan).

### Cell culture

(CAGA)_9_-Luc CCL64 cells were kindly provided by Prof. Hideaki Kakeya (Kyoto University, Kyoto, Japan), the hepatic stellate cell line LX-2 was kindly provided by Prof. Norifumi Kawada (Osaka City University, Osaka, Japan), and the human hepatoma cell line Huh-7.5.1 were maintained in Dulbecco's modified Eagle's medium (DMEM) supplemented with 10% fetal bovine serum, penicillin, and streptomycin. HC cells, a normal human hepatocyte cell line purchased from Cell Systems (Kirkland, WA), were cultured in CS-C complete medium (Kirkland, WA).

### Protein preparation

The N-terminal histidine or 3xFLAG-tagged NS3 protease, and the extracellular domain of human TβRI and TβRII were expressed in *Escherichia coli* by isopropyl-β-thiogalactopyranoside induction. The protein was purified by affinity chromatography in a HisTrap HP column (GE Healthcare, Waukesha, WI). Detailed procedures are in the Supplementary information.

### Enzyme-linked immunosorbent assay (ELISA)

TGF-β2 ELISA was performed using a TGF-β2 Emax® Immune Assay System ELISA kit (Promega, Madison, WI) according to the manufacturer's instructions.

### Luciferase assay

The mink lung epithelial cell line CCL64, which stably expressed (CAGA)_9_-MLP-luciferase and contained nine copies of a Smad-binding CAGA box element upstream of a minimal adenovirus major late promoter (2 × 10^4^ cells/well)^23^, was seeded into 96-well plates. The next day, the medium was replaced with fresh medium containing 0.1% bovine serum albumin, and the cells were cultured for an additional 24 hours. The cells were extracted with lysis buffer, and luciferase activity was measured by a Luciferase Assay System (Promega, Madison, WI) according to the manufacturer's instructions.

### Real-time RT-PCR

The isolation of total RNA and real-time RT-PCR were performed as described previously^24^. Briefly, total RNA was extracted using the RNeasy mini kit (Qiagen, Valencia, CA) according to the manufacturer's protocols. RNA (0.5 μg) was reverse transcribed to cDNA using the PrimeScript® RT Master Mix (Takara Bio Inc., Shiga, Japan). The mRNA expression levels were determined using real-time PCR. Real-time PCR was performed with the Thermal Cycler Dice® Real Time System, using the SsoAdvanced™ SYBR® Green Supermix (Bio-Rad Laboratories, Hercules, CA) and normalized to GAPDH mRNA expression. The primer sequences used were as follows: human TGF-β1 forward: 5′-ACT ATT GCT TCA GCT CCA CGG A-3′, reverse: 5′-GGT CCT TGC GGA AGT CAA TGT A-3′; human collagen α1 (I) forward: 5′-ACG AAG ACA TCC CAC CAA TC-3′, reverse: 5′-AGA TCA CGT CAT CGC ACA AC-3′; human GAPDH forward: 5′-GGA GTC AAC GGA TTT GGT-3′, reverse: 5′-AAG ATG GTG ATG GGA TTT CCA-3′; and human TβRI forward: 5′-CTT AAT TCC TCG AGA TAG GC-3′, reverse: 5′-GTG AGA TGC AGA CGA AGC-3′.

### Immunofluorescence staining

The cells were grown on eight-well chamber slides or glass bottom dishes and were incubated with HCV virion for 24 hours at 37°C. The cells were washed with PBS, fixed with 4% paraformaldehyde for 10 min at room temperature, and permeabilized with 0.1% Triton X-100 for 20 min at room temperature. After blocking with 3% BSA/10% normal goat serum/PBS for 30 min, the cells were incubated with primary antibodies for 2 hours, followed by incubation with secondary antibodies for 30 min at RT. For detecting NS3 and TβRI on the cell surface, the cells were fixed without permeabilization after incubation with the secondary antibodies. After being washed with PBS, the cells were mounted with Vectashield DAPI mounting medium (Vector Laboratories, Inc., Burlingame, CA) and observed under a Zeiss LSM 700 laser scanning confocal microscope. For quantitative fluorescence analyses, the intensity of phosphorylated Smad3 and the colocalization of NS3 and TβRI (Pearson's colocalization coefficient values) in each panel were calculated with ZEN software.

### Proximity ligation assay (PLA)

HCV-infected Huh-7.5.1 cells were fixed with 4% paraformaldehyde for 10 min at room temperature and subjected to in situ PLA using a Duolink in situ red starter kit (Olink Bioscience, Uppsala, Sweden) according to the manufacturer's instructions. Briefly, cells were blocked and incubated with primary antibodies against NS3 and TβRI, followed by incubation with the PLA probes, which were secondary antibodies (anti-mouse and anti-rabbit) conjugated to oligonucleotides. DNA ligase was added to enable the formation of circular DNA strands when the PLA probes were in close proximity. This step was followed by incubation with oligonucleotides and polymerase for rolling circle amplification^25^. Texas red-labeled oligonucleotides, which hybridize to the amplified products, were used for visualization. The cells were observed under a Zeiss LSM 700 laser scanning confocal microscope.

### Immunoprecipitation and immunoblotting

Anti-FLAG M2 affinity beads were pretreated with 5% bovine serum albumin in 20 mM Tris-HCl, pH 7.5, 150 mM NaCl overnight. Isotype control IgG was bound to Protein G PLUS-Agarose (Santa Cruz) pretreated with 5% bovine serum albumin in 20 mM Tris-HCl, pH 7.5, 150 mM NaCl. Cell lysates with 3xFLAG or 3x-FLAG-NS3 (2 mg protein) were incubated with 50 μl of the beads (10% slurry) at 4°C for 3 hours. The beads were then washed three times with the lysis buffer and incubated with lysates containing 6xHis-TβRI or 6xHis-TβRII (0.5 mg protein) at 4°C overnight. The bound proteins were eluted with the SDS-PAGE sample buffer after washing four times with the lysis buffer and then were subjected to SDS-PAGE (15% acrylamide) followed by transfer onto a PVDF membrane (Pall). The proteins were then visualized using anti-His tag HRP DirectT (MBL, 1/5000) or anti-FLAG BioM2 antibody (Sigma, 10 μg/ml) and horseradish peroxidase-conjugated anti-biotin antibody (Cell Signaling) using the ECL Western blotting detection reagent (GE Healthcare).

### In silico docking simulation

The protein-protein docking simulation was implemented based on the geometric complementarity^26^ between NS3 protease (PDB ID, 1NS3) and TβRI (PDB ID, 2PJY). Specifically, coordinates of the proteins were projected onto three-dimensional grids separated from each other at regular intervals. A surface score and an intramolecular score were assigned to each grid. This operation was conducted for both the receptor and the ligand. Next, convolution between the obtained grids was performed, the surfaces were explored exhaustively, and the complementarities of the binding states were calculated based on the scores. Amino acid residues appearing frequently in binding states with high complementarity scores can be estimated to be residues that are highly likely to appear in the interaction with an actual receptor. Accordingly, amino acid residues with an interatomic distance of 3.8 Å or less in the putative binding states were defined as contact residues and regarded as the putative contact residues of NS3 and TβRI.

### Animal experiment

Chimeric mice with humanized livers were generated as previously described using urokinase-type plasminogen activator (uPA)-transgenic/SCID mice^27^. All mice were transplanted with frozen human hepatocytes obtained from a single donor. All animal experiments were approved by RIKEN Institutional Animal Use and Care Administrative Advisory Committees and were performed in accordance with RIKEN guidelines and regulations. Infection, extraction of serum samples, and euthanasia were performed under isoflurane anesthesia. Male chimeric mice (12- to 14-week old) were intravenously injected with 100 μl HCV J6/JFH-1 strain (1 × 10^8^ copies/ml). Four weeks after HCV inoculation, anti-NS3 antibodies against predicted binding sites with the TβRI receptor were administered at doses of 0.5 mg/kg of BW or 5 mg/kg of BW twice a week for twelve weeks. Normal mouse IgG was administered at a dose of 5 mg/kg of BW as a control. When the animals were euthanized, the livers were either fixed with 4% paraformaldehyde for histological analysis or frozen immediately in liquid nitrogen for mRNA isolation.

### Staining of liver tissue sections

The liver tissues were fixed in 4% paraformaldehyde and embedded in paraffin, and tissue sections (6 μm in thickness) were prepared with a Leica sliding microtome (Leica Microsystems, Nussloch, Germany). The liver tissue sections were deparaffinized, rehydrated, and incubated for 5 min with a drop of Proteinase K (Dako Envision) in 2 mL of 0.05 M Tris-HCl buffer (pH 7.5) at room temperature. The liver tissue sections were stained with Mayer's hematoxylin solution (Muto Chemicals) and 1% eosin Y solution (Muto Chemicals). Sirius Red, which results in a red staining of all fibrillar collagen, was used to evaluate fibrosis. Briefly, the liver sections were stained with 0.05% Fast Green FCF (ChemBlink, Inc. CAS: 2353-45-9) and 0.05% Direct Red 80 (Polysciences, Inc. CAS: 2610-10-18) in saturated picric acid (Muto Chemicals) for 90 min at room temperature. The ratios of Sirius Red positive/total area (%) from 6 randomly selected fields were measured for each group using WinROOF software (Mitani Corp., Tokyo, Japan).

### Statistics

Statistical analysis was performed using one-way analysis of variance, followed by Dunnett's post-hoc test. A two-tailed Student's *t*-test was used to evaluate differences between the two groups. The Kruskal-Wallis test followed by Dunn's post-hoc test was used for multiple comparisons of Sirius Red positive areas.

## Author Contributions

Sakata K., Hara M. and Yaguchi S. performed experiments. Sakata K., Matsuura T., Miyazawa K., Imoto M. and Kojima S. wrote the manuscript. Terada T., Matsumoto T., Shirouzu M., Yokoyama S., Yamaguchi T. and Suzuki T. contributed to the production and the purification of recombinant NS3 and its antibodies. Watanabe N., Aizaki H. and Wakita T. contributed to the production and the purification of HCV and discussion from the point of view of virology. Takaya D. performed docking simulation to predict binding sites. Sakata K. and Kojima S. planned the research. Kojima S. supervised the entire project.

## Supplementary Material

Supplementary Information Supplementary Information

## Figures and Tables

**Table 1:** The amino acid sequences of predicted binding sites between NS3 protease and TβRI

	NS3 protease	TβRI
Site 1	TGRDKNQVEGEVQVVSTATQS	FVSVTETTDKVIHNSM
Site 2	TNVDQDLVGWPAPPGARSLTP	IAEIDLIPRDRPFV
Site 3	GDNRGSLLSPRPVSYLKGSS	CAPSSKTGSVTTTY

The underlined letters denote the putative contact residues.
