# Supplementary material for: HCV NS3 protease enhances liver fibrosis via binding to and activating TGF-β type I receptor
Source: Sci Rep. 2013 Nov 22;3:3243. doi: 10.1038/srep03243 (PMC3837337; doi:10.1038/srep03243)
Supplement: Supplementary Information [file srep03243-s1.pdf]

HCV NS3 protease enhances liver fibrosis via binding to and activating TGF-β type I receptor

Kotaro Sakata[1,2,3], Mitsuko Hara[1], Takaho Terada[4,5], Noriyuki Watanabe[6], Daisuke Takaya[4,7], So-ichi Yaguchi[8], Takehisa Matsumoto[4,7], Tomokazu Matsuura[9], Mikako Shirouzu[4,7], Shigeyuki Yokoyama[4,5], Tokio Yamaguchi[10], Keiji Miyazawa[8], Hideki Aizaki[6], Tetsuro Suzuki[11], Takaji Wakita[6], Masaya Imoto[2], and Soichi Kojima[1,*]

[1]Micro-signaling Regulation Technology Unit, RIKEN Center for Life Science Technologies, Saitama 351-0198, Japan

[2]Department of Biosciences and Informatics, Faculty of Science and Technology, Keio University, Kanagawa 223-8522, Japan

[3]Drug Discovery Laboratory, Wakunaga Pharmaceutical Co., Ltd., Hiroshima 739-1195, Japan

[4]RIKEN Systems and Structural Biology Center, Kanagawa 230-0045, Japan

[5]RIKEN Structural Biology Laboratory, Kanagawa 230-0045, Japan

[6]Department of Virology II, National Institute of Infectious Diseases, Tokyo 162-8640, Japan

[7]Division of Structural and Synthetic Biology, RIKEN Center for Life Science Technologies, Kanagawa 230-0045, Japan

[8]Department of Biochemistry, Interdisciplinary Graduate School of Medicine and Engineering,

University of Yamanashi, Yamanashi 409-3898, Japan

[9]Department of Laboratory Medicine, the Jikei University School of Medicine, Tokyo 105-8461, Japan

[10]RIKEN Program for Drug Discovery and Medical Technology Platforms, Saitama 351-0198, Japan

[11]Department of Infectious Diseases, Hamamatsu University School of Medicine, Shizuoka 431-3192, Japan

Supplementary Information

Supplementary Methods

Protein preparation

The gene-encoding N-terminal histidine-tagged NS3 protease was cloned into the pET32a (+) vector and expressed in *Escherichia coli* (KRX) by isopropyl-β-thiogalactopyranoside induction. The protein was purified by affinity chromatography in a HisTrap HP column (GE Healthcare, Waukesha, WI) and was dialyzed in 20 mM Tris-HCl buffer (pH 8.0) containing 500 mM NaCl, 20 μM $ZnCl_2$, and 1 mM Tris (2-carboxyethyl) phosphine. p3xFLAG-2 was constructed from pFLAG2 (Sigma) by inserting a synthetic oligonucleotide between the *Hin*dIII and *Eco*RI sites. The cDNA sequence-encoding NS3 protease (1-181) was amplified by PCR using pcDNA3-MEF-NS3-4A as a template and was cloned into the *Eco*RI and *Xho*I sites of p3xFLAG-2. The cDNA sequences encoding the extracellular domain of human TβRI (30 to 115) and TβRII (15 to 136) were amplified by PCR and were cloned into the *Bam*HI and *Xho*I sites of pQE30 (Qiagen) or pGEX-6P-1 (GE Healthcare). p3xFLAG-NS3 was introduced into the *E. coli* strain BL21(DE3) harboring pG-KJE8 (TAKARA). pQE-TβRI and pQE-TβRII were introduced into *E. coli* M15[pREP4] harboring pG-KJE8. pGEX-TβRI and pGEX-TβRII were introduced into *E. coli* BL21 harboring pG-KJE8 (TAKARA). The proteins were expressed overnight at 20°C after induction with 0.5 mM

isopropyl-β-thiogalactopyranoside. After being harvested, the cells were lysed in a buffer solution (20 mM Tris-HCl, pH 7.5, 150 mM NaCl, 20 μM $ZnCl_2$) by sonication on ice. The lysate was clarified by centrifugation at 4°C, 10,000 x *g* for 20 min.

GST pull-down assay with blocking peptides

Cell lysates with GST-TβRI or GST (0.5 mg protein) incubated with 50 μg of the peptides (NS-1, NS-2, or NS-3) on ice for 3 hours. Cell lysates with 3xFLAG-NS3 (0.3 mg) were then added and further incubated on ice for 2 hours. After centrifugation for 20 min, the supernatants were incubated with 20 μl of Glutathione Sepharose beads (10% slurry) at 4 ºC for 1 hour. Beads were washed four times with 20 mM Tris-HCl, pH 7.5, 150 mM NaCl, 20 μM $ZnCl_2$. Bound proteins were eluted with 20 mM glutathione in 20 mM Tris-HCl, pH 7.5, 150 mM NaCl, and visualized by immunoblotting using anti-FLAG M2 antibody (Sigma) or anti-GST antibody (GE Healthcare). Secondary antibodies used are as follows: anti mouse IgG-HRP (Jackson), anti-goat IgG-HRP (Wako).

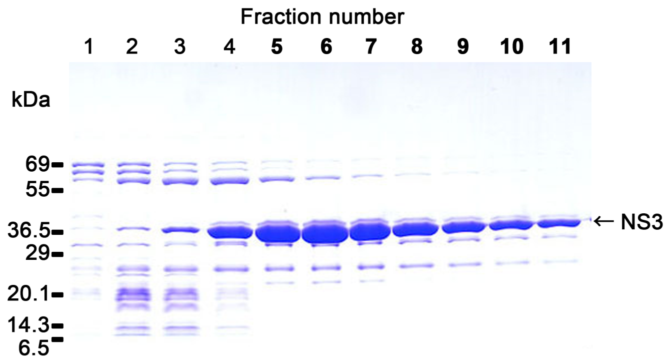

Supplementary Figure S1. SDS-PAGE profile of the NS3 protein. Bacterially expressed NS3 protein was sonicated 10 times for 30 seconds and centrifuged at 16,000 x *g* for 20 min. The supernatant was filtrated and fractionated by a HisTrap HP column. Fraction numbers 5 to 11 were mixed and used for the subsequent assays.

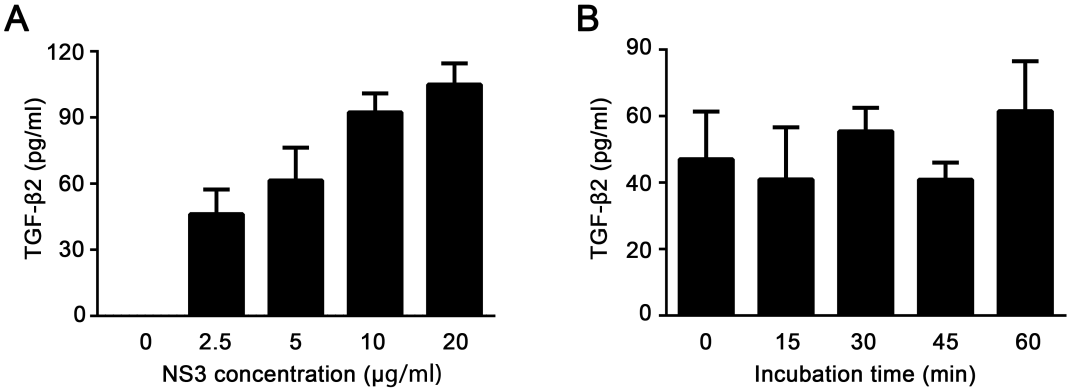

Supplementary Figure S2. NS3 did not activate TGF-β2. (A) The indicated concentrations of recombinant NS3 protease were incubated with conditioned medium obtained from HEK293T cells transiently overexpressing latent TGF-β2 at 37°C for 30 min. The reaction mixture was subjected to active TGF-β2 ELISA. (B) 10 μg/ml recombinant NS3 protease was incubated with conditioned medium obtained from HEK293T cells transiently overexpressing latent TGF-β2 at 37°C for the indicated times. The reaction mixture was subjected to active TGF-β2 ELISA.

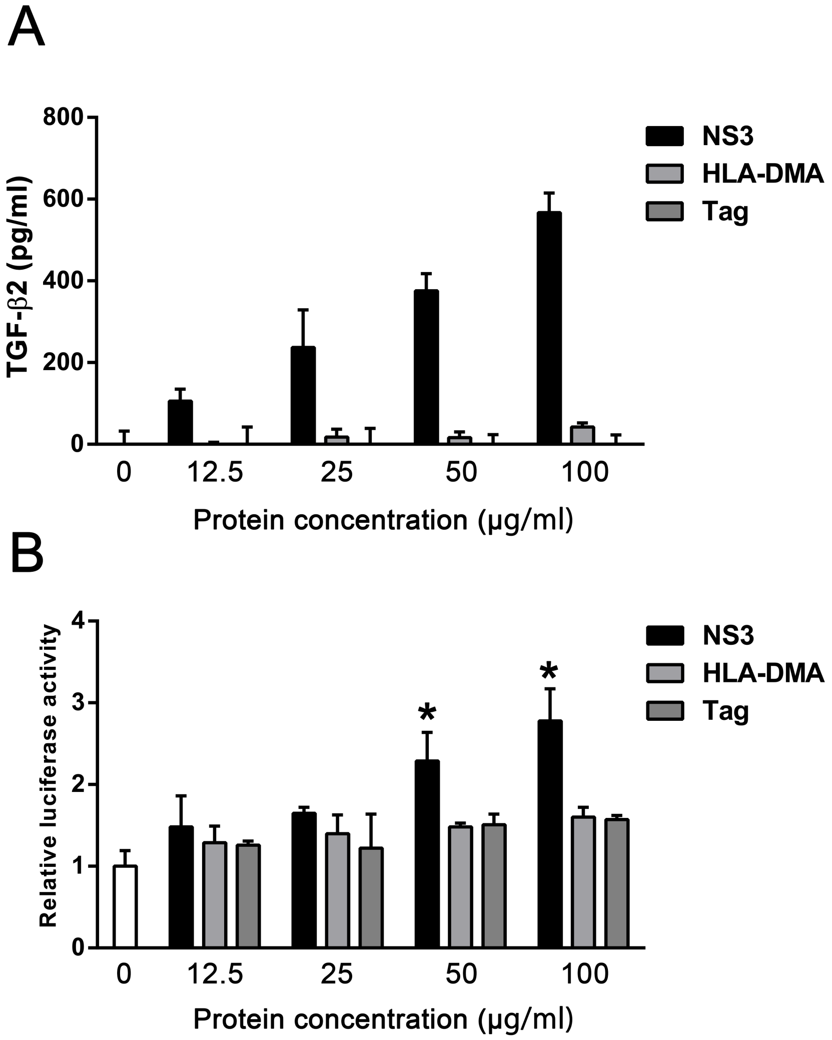

Supplementary Figure S3. NS3 specifically exerted TGF-β mimetic activity. (A) The specific TGF-β2 antigenicity of NS3. Recombinant NS3 protease (NS3, black bar), HLA class II histocompatibility antigen, DM α chain (HLA-DMA, gray bar), and fractions purified from carrier-free plasmid samples (Tag control, white bar) were subjected to TGF-β2 ELISA. (B) The specific TGF-β bioactivity of NS3. $(CAGA)_9$-Luc CCL64 cells were stimulated with the indicated concentrations of recombinant NS3 protease, HLA-DMA, and tag control samples for 24 hours. After 24 hours, the cells were harvested and luciferase activity measured. † $p < 0.05$ compared with untreated control cells.

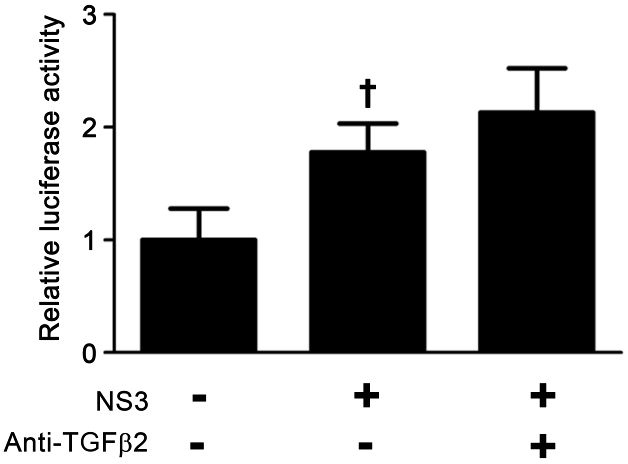

Supplementary Figure S4. TGF-β mimetic activity of NS3 was not inhibited by anti-TGF-β2 antibody. $(CAGA)_9$-Luc CCL64 cells were stimulated with 100 μg/ml of recombinant NS3 protease for 24 hours, with or without anti-TGF-β2 antibody used for TGF-β2 ELISA. After 24 hours, the cells were harvested and luciferase activity measured. †$p < 0.05$ compared with untreated control cells. The data are shown as the mean ± SD (n=3).

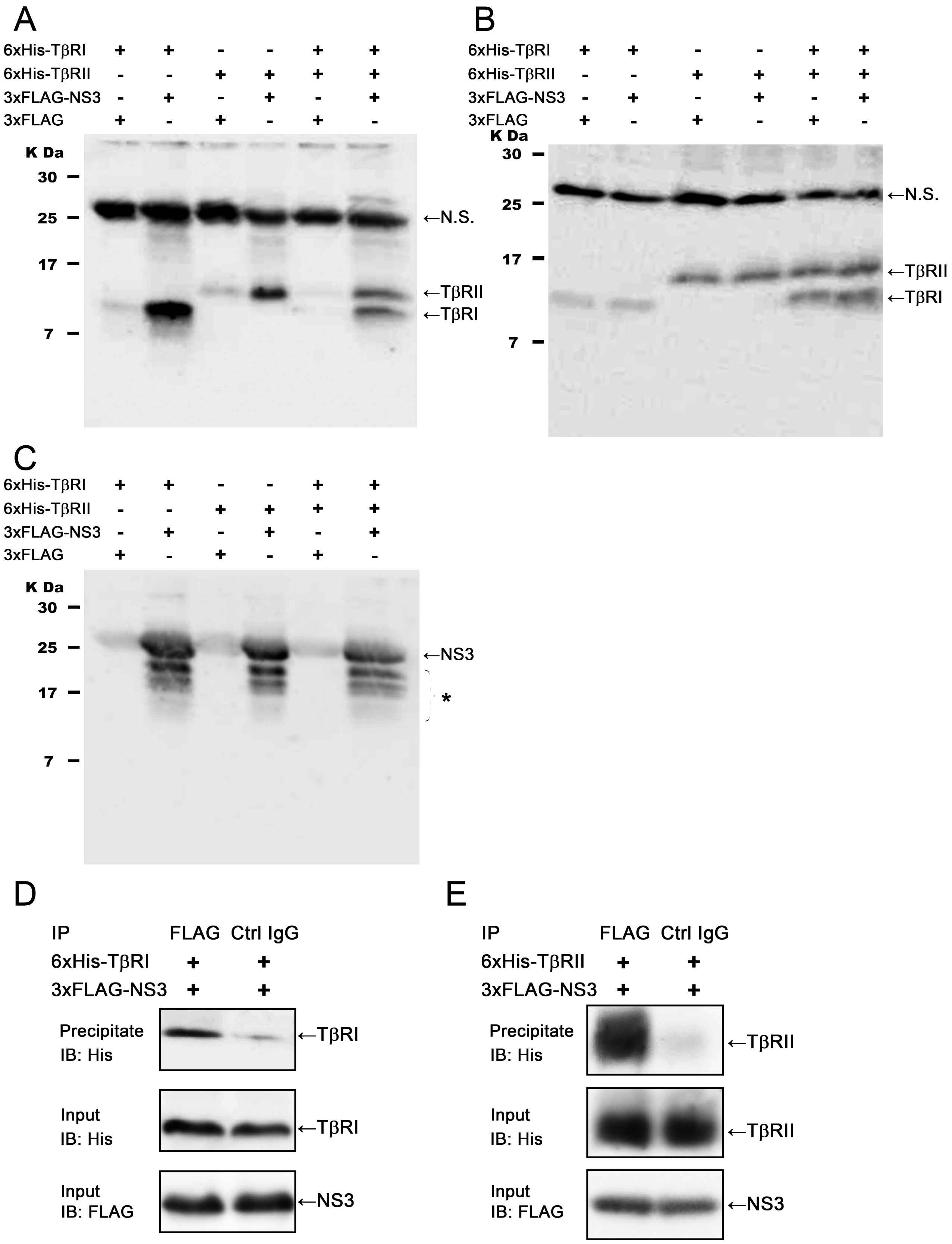

Supplementary Figure S5. Physical interaction of NS3 protease with TβRI and TβRII.

FLAG-tagged NS3 protease was incubated with 6xHis-tagged TβRI and/or TβRII and

immunoprecipitated by anti-FLAG antibody (Panels A-C and left lanes in panels D and E) or isotype control IgG (Ctrl IgG) (right lanes in panels D and E). The coprecipitated proteins were visualized by immunoblotting using anti-His antibody (Panel A and the upper panel of D and E). Input samples before co-immunoprecipitation were visualized by immunoblotting using anti-His antibody (B and the middle panel of D and E) and anti-FLAG antibody (C and the lower panel of D and E). The gels were run under the same experimental conditions. N.S. refers to a non-specific band, and the asterisk indicates degradation fragments.

```
                                          Site 1
                                  TGRDKNQVEGEVQVVSTATQS
1a    1 APITAYAQQTRGLLGCIITSLTGRDKNQVEGEVQIVSTATQTFLATCINGVCWTVYHGAGTRTIASPKGP  70
1b    1 ......SR..........................V......S.....V............SK.L.G....  70
2a    1 ...............A.VV.M....RTEQA.....L..VS.S..G.T.S..L........NK.L.GLR..  70
2b    1 ......T........A.VV........EQA.Q..VL.SV..S..G.S.S..L........NK.L...R..  70

              Site 2                                          Site 3
        TNVDQDLVGWPAPPGARSLTP                          GDNRGSLLSPRPVSYLKGSS
1a   71 VIQMYTNVDQDLVGWPAPQGSRSLTPCTCGSSDLYLVTRHADVIPVRRRGDSRGSLLSPRPISYLKGSSG 140
1b   71 IA................P.A..............................N.........V........ 140
2a   71 .T...SSAEG......S.P.TK..E..K..AV.......N.....A.....K..A........T...... 140
2b   71 .T....SAEG......S.P.TK..D.....AV.......N........KD.R..A......L.T...... 140

1a  141 GPLLCPAGHAVGLFRAAVCTRGVAKAVDFIPVENLGTTMRS                             181
1b  141 ......S.....V................V...SME.....                             181
2a  141 ..V...R..V.........S.....SI......T.DVVT..                             181
2b  141 ..V...R............A.....SI......S.DIAR.T                             181

                                                          Catalytic triad
```

Supplementary Figure S6. Amino acid alignment of HCV NS3 protease (1-181) of various genotypes, including 1a (H77 strain), 1b (HCV-N strain), 2a (JFH-1 strain), and 2b (MA strain). The multiple sequence alignments were generated by GENETYX version 11 (Genetyx, Tokyo, Japan). The following amino acid sequences of HCV NS3 protease (1-181) of various genotypes were obtained from GenBank: AAB67036.1 for genotype 1a (H77 strain); AAB27127.1 for genotype 1b (HCV-N); AEJ86546.1 for genotype 2a (JFH-1 strain); and BAB08107.1 for genotype 2b (MA strain). The dots indicate amino acids that are identical to the top sequence. Sites 1, 2, and 3 are the predicted binding sites with TβRI. The amino acids enclosed in square boxes indicate the catalytic triad of NS3 protease. The predicted binding sites, particularly site 3, were conserved among genotypes.

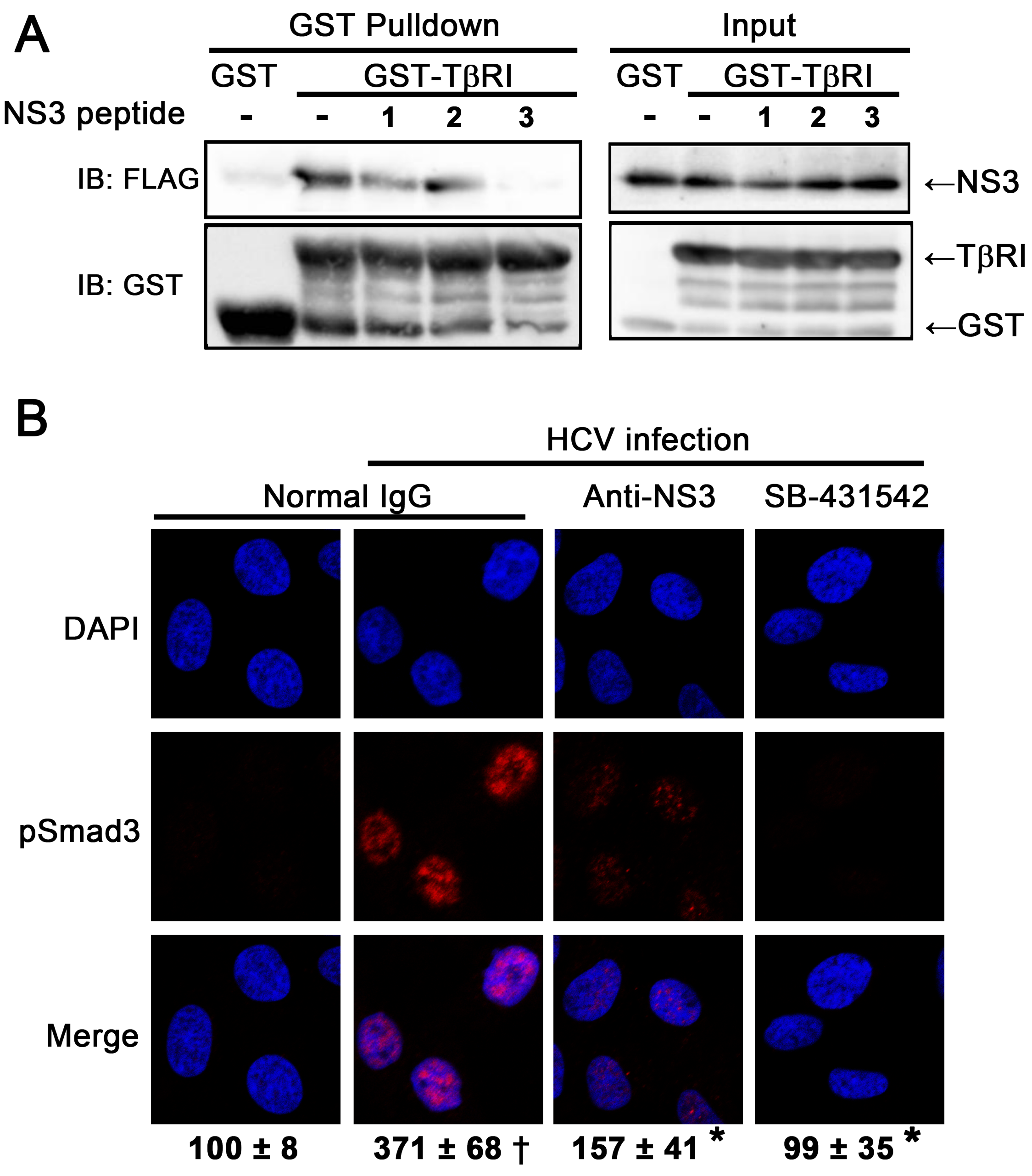

Supplementary Figure S7. Inhibition assays using NS3 peptides derived from the predicted binding sites to TβRI, and anti-NS3 antibody. (A) Inhibition by the NS3 peptides of physical interaction between NS3 protease with TβRI. GST-TβRI or GST was pre-incubated

with indicated peptides for 3 hours, then mixed with 3xFLAG-NS3, followed by pull-down with Glutathione-Sepharose. Bound proteins were eluted with 20 mM glutathione and subjected to immunoblotting with anti FLAG antibody (NS3 protein) or anti-GST antibody (GST-fusion proteins) (left panel). Input samples before pull-down were also visualized by immunoblotting (right panel). The gels have been run under the same experimental conditions. (B) Inhibition of HCV-induced Smad3 phosphorylation in Huh-7.5.1 cells by anti-NS3 antibody. The cells were fixed and stained with DAPI and anti-phospho-Smad3 antibody, as described in the Materials and Methods section in the main text. SB-431542, a TβRI kinase inhibitor, was used as a positive control. The relative fluorescence intensities (% of non-infected control cells) from 4 randomly selected fields from each dish were calculated by the ZEN software and are shown as the mean ± SD. †$p < 0.05$ compared with non-infected cells. *$p < 0.05$ compared with HCV-infected normal IgG-treated cells. The results are representative of two independent experiments with similar results.

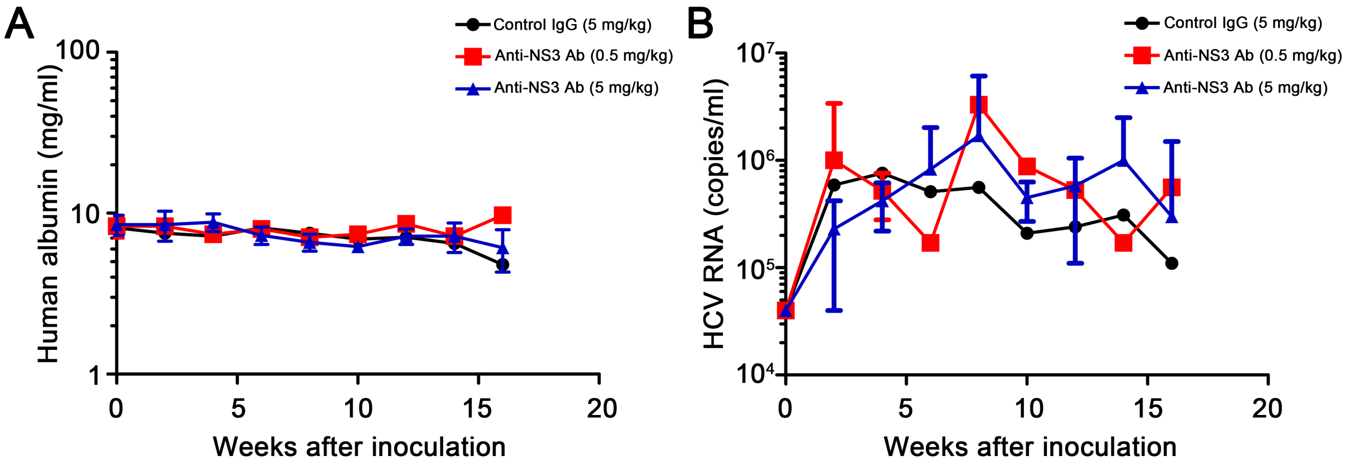

Supplementary Figure S8. Anti-NS3 antibody did not affect human albumin concentrations in chimeric mouse blood and HCV RNA levels in HCV-infected chimeric mice. Blood samples were collected every week from the orbital veins at the indicated times. (A) Human albumin concentrations were measured using an Alb-II kit (Eiken Chemical, Tokyo, Japan) according to the manufacturer's instructions. The data are shown as the mean ± SD. (B) Total RNA was extracted from serum using a SepaGene RV-R RNA extraction kit (Sanko Junyaku, Tokyo, Japan). The amplification of HCV RNA was performed using TaqMan EX RT-PCR Core Reagents (Life Technologies, Carlsbad, CA). The primer sequences used are as follows: HCV RNA forward: 5'- CGG GAG AGC CAT AGT GG-3'; reverse: 5'- AGT ACC ACA AGG CCT TTC G-3'; and probe: 5'-FAM- CTG CGG AAC CGG TGA GTA CAC-TAMRA-3'. The data are shown as the geometric mean ± SD.

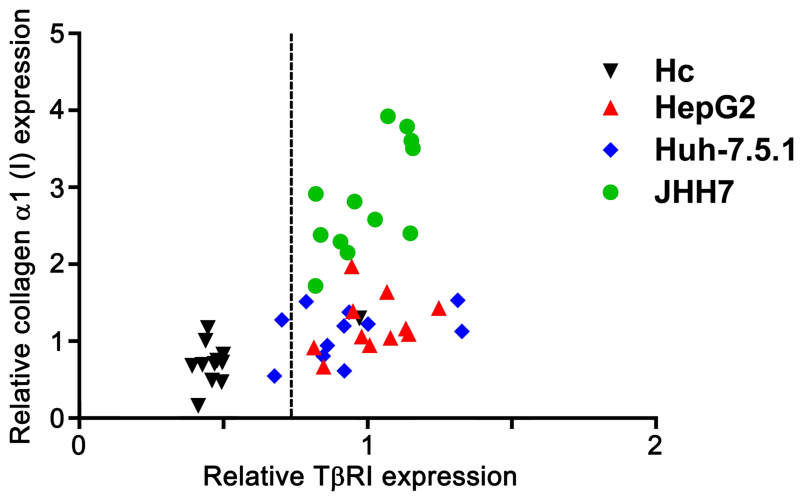

Supplementary Figure S9. Correlation between collagen α1 (I) and TβRI expression in hepatic cell lines. Various human hepatic cell lines, including Hc, HepG2, Huh-7.5.1, and JHH7, were treated with or without 25 μg/ml NS3 for 12 hours, and the mRNA expression levels of TβRI, collagen α1 (I), and glyceraldehyde-3-phosphate dehydrogenase (GAPDH) were measured as described in the Materials and Methods section in the main text. The relative expression levels of TβRI (x-axis) and collagen α1 (I) (y-axis) were normalized to GAPDH. The broken line shows the predicted threshold.
